# *CXCR4* promotes gefitinib resistance of Huh7 cells by activating the c‐Met signaling pathway

**DOI:** 10.1002/2211-5463.13305

**Published:** 2021-10-19

**Authors:** Dali Zhao, Zhiqiang Yang, Chen Chen, Zhipeng Zhang, Yangsheng Yu, Zhituo Li

**Affiliations:** ^1^ Department of General Surgery The First Affiliated Hospital of Harbin Medical University China

**Keywords:** *Cav‐1*, c‐Met, *CXCR4*, gefitinib resistance, hepatocellular carcinoma

## Abstract

C‐X‐C chemokine receptor type 4 (*CXCR4*) expression is associated with poor prognosis of hepatocellular carcinoma (HCC). The aim of this study was to explore the biological role of *CXCR4* in gefitinib resistance of HCC. Compared with a normal, non‐gefitinib‐resistant, human HCC cell line (Huh7), *CXCR4* mRNA and protein were highly expressed in gefitinib‐resistant Huh7 cells (Huh7‐R). Cell proliferation was decreased, and apoptosis was enhanced in Huh7 cells in the presence of gefitinib. These influences conferred by gefitinib treatment on proliferation and apoptosis of Huh7 cells were abolished by *CXCR4* overexpression. *CXCR4* knockdown reduced the proliferation ability of HuH‐7R cells after gefitinib treatment. Importantly, *CXCR4* overexpression had no influence on caveolin 1 (*Cav‐1*) expression; similarly, *Cav‐1* silencing did not cause a substantive change in *CXCR4* expression. However, *CXCR4* activated *Cav‐1*, c‐Met, and Raf‐1 in Huh7 cells, whereas *Cav‐1* silencing repressed the expression of Raf‐1 and phosphorylated c‐Met in Huh7 cells. *CXCR4* overexpression promoted proliferation and repressed apoptosis in gefitinib‐treated Huh7 cells, which was partly rescued by PHA‐665752 (a c‐Met inhibitor) treatment or c‐Met deficiency. Finally, we constructed a tumor xenograft model to determine the influence of *CXCR4* overexpression on tumor growth of HCC. *CXCR4* overexpression accelerated tumor growth of HCC, which was abrogated by c‐Met deficiency. These findings demonstrate that *CXCR4* overexpression activates c‐Met via the *Cav‐1* signaling pathway, thereby promoting gefitinib resistance of Huh7 cells. Thus, this study highlights novel insights into the mechanism of gefitinib resistance of HCC and *CXCR4* may become a potential target for HCC treatment.

## Graphical abstract

C‐X‐C chemokine receptor type 4 (*CXCR4*) expression is associated with poor prognosis of hepatocellular carcinoma. This work demonstrates that *CXCR4* overexpression activates c‐Met via the caveolin 1 signaling pathway, thereby promoting gefitinib resistance of Huh7 cells. Thus, this study suggests that *CXCR4* may be a potential target for hepatocellular carcinoma treatment.

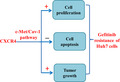

**Abbreviations**
Cat: catalog number
*Cav‐1*
: caveolin 1
CCK‐8: Cell Counting Kit‐8
Ctrl: control
*CXCR4*
: C‐X‐C chemokine receptor type 4
DMEM: dulbecco's modified eagle medium
EGFR: epidermal growth factor receptor
ERBB3: epidermal growth factor receptor 3
FBS: foetal bovine serum
HCC: hepatocellular carcinoma
HGF: hepatocyte growth factor
LV: lentivirus
NC: negative control
PI: propidium iodide
PI3K: phosphatidylinositol 3‐kinase
qRT‐PCR: quantitative real‐time PCR
sh: short hairpin
si: small interference
TKI: tyrosine kinase inhibitor
WB: western blot

Hepatocellular carcinoma (HCC) is a highly heterogeneous malignant tumor with a high morbidity and mortality [1]. HCC is insidious and develops rapidly. Many patients have progressed to the middle and late stages of HCC when they are discovered. Therefore, patients with HCC often lose the opportunity to undergo radical surgery. So far, although scholars have made many achievements and new progress in the research of HCC, the pathogenesis of HCC is still unclear. The molecular mechanism of HCC may be related to the abnormal cell signal transduction pathways, changes in the interstitial microenvironment, and disorders of tumor‐related genes [2]. Clinically, HCC therapy includes surgical resection, interventional chemotherapy, radiotherapy, biological therapy, and minimally invasive treatment. In recent years, molecular targeted therapy has become a research focus [3].

Epidermal growth factor receptor (EGFR) belongs to a member of the receptor tyrosine kinase superfamily, which is expressed in various malignant tumors. The ligand forms a complex with EGFR to activate tyrosine kinases and signal transduction pathways and thus regulates cell proliferation, invasion, metastasis and antioxidation [4]. EGFR tyrosine kinase inhibitor (TKI) drugs, such as gefitinib and erlotinib, have been used in clinical practice [5]. However, some patients are not sensitive to EGFR‐TKIs or develop drug resistance during treatment, which directly affects the clinical efficacy of EGFR‐TKIs [6]. In addition to the partial secondary mutations of EGFR, many molecules located in the downstream signaling pathways or subactivation pathways of EGFR are also involved in the formation of drug resistance, such as the c‐Met signaling pathway [7, 8]. Amplification or up‐regulation of c‐Met may participate in the mechanisms of EGFR‐TKI resistance. c‐Met is a transmembrane receptor for hepatocyte growth factor (HGF) with autonomous phosphorylation activity, and it is a member of the receptor tyrosine kinases superfamily [9]. The combination of HGF and c‐Met promotes autophosphorylation of tyrosine residues in the cytoplasm, which in turn activates the protein tyrosine kinase domain in the cytoplasm. The activated protein tyrosine kinase causes autophosphorylation of tyrosine at the carboxyl terminus of c‐Met. Subsequently, various substrate proteins in the cytoplasm are rapidly phosphorylated. The activated signaling pathways avoid the damage of TKI to cells, promote proliferation of cancer cells, and ultimately lead to the resistance of patients with cancer to EGFR‐TKIs [10]. However, the molecular mechanism of EGFR‐TKIs in regulating c‐Met expression in HCC is still unclear.

C‐X‐C chemokine receptor type 4 (*CXCR4*) is highly expressed in HCC cells, and the up‐regulation of *CXCR4* is closely associated with the poor prognosis of HCC . This study has suggested that *CXCR4* may be involved in the regulation of EGFR‐TKIs resistance in HCC. A previous study has reported that *CXCL12*/*CXCR4* induces c‐Met activation by activating the caveolin 1 (*Cav‐1*) signaling pathway [11]. *Cav‐1* and c‐Met are colocalized in HCC cells, and the reciprocal activating crosstalk between c‐Met and *Cav‐1* promotes invasive phenotype in HCC by promoting the oncogenic signaling of c‐Met [12]. Overexpression of *Cav‐1* initiates carcinogenesis in liver cirrhosis and subsequently contributes to the progression of HCC [13]. *Cav‐1* accelerates invasion and metastasis of HCC, which attributes to up‐regulate the expression of Pofut1 [14]. Moreover, a previous study has confirmed that *Cav‐1* inhibition enhances the sensitivity to gefitinib in lung adenocarcinoma cells. Thus, we speculated whether *CXCR4* can enhance EGFR‐TKI (gefitinib) resistance in HCC by activating the *Cav‐1*/c‐Met signaling pathway. This work conducted research on this hypothesis.

## Materials and methods

### Cell culture

A human HCC cell line (Huh7) was obtained from Chinese Academy of Sciences (Shanghai, China). Huh7 cells were cultured in dulbecco's modified eagle medium (DMEM) (catalog number [Cat]: 11966025; Gibco, Carlsbad, CA, USA) containing 10% foetal bovine serum (FBS) (Cat: 16140063; Gibco) and 1% penicillin/streptomycin (Cat: P1400; Solarbio, Beijing, China). Huh7 cells were incubated at 37 °C and 5% CO_2_. Huh7 cells were treated with 10 μm gefitinib (AstraZeneca, Wilmington, DE, USA, and Macclesfield, UK) or combined with 1 μm PHA‐665752 (c‐Met inhibitor; Cat: HY‐11107; MedChem Express, Monmouth Junction, NJ, USA) for 48 h.

Gefitinib‐resistant Huh7 cells (Huh7‐R cells) were induced by treating with a gradual gradient of gefitinib concentration. Cells were initially treated with 10 nm gefitinib, until the cells could grow stably in the DMEM containing 10 μm gefitinib. Two weeks before the experiment, Huh7‐R cells were cultured in DMEM without gefitinib for further use. Huh7‐R cells were treated with 10 μm gefitinib for 48 h.

### Cell transfection

The full length of *CXCR4* was subcloned into the pcDNA3.1 vector, pcDNA3.1‐CXCR4, (GeneChem, Shanghai, China). The negative control (NC) vector pcDNA3.1‐NC served as control (Ctrl). Small interference (si) RNA specifically targeting *CXCR4* (si‐CXCR4), *Cav‐1* (si‐Cav‐1) or c‐Met (si‐c‐Met) and the corresponding Ctrl (si‐Ctrl) were purchased from GeneChem. Lentivirus (LV) harboring *CXCR4* (LV‐CXCR4) or short hairpin (sh) RNA specifically targeting c‐Met (LV‐sh c‐Met) and the corresponding NC (LV‐NC) were generated by GeneChem. Huh7 and Huh7‐R cells (100 μL) were transfected with 75 ng plasmids using 3 μL HiPerFect Transfection Reagent (Cat: 301704; Qiagen, Hilden, Germany). The transfected Huh7 cells were incubated at 37 °C and 5% CO_2_ for 48 h.

### Tumor xenograft experiments

Male BALB/c nude mice, 4–6 weeks old (weighing 15–20 g), were purchased from Vital River Laboratory Animal Technology (Beijing, China). These mice were housed under specified pathogen‐free conditions with an optimal temperature (22–24 °C) and humidity (40–60%) and a 12‐h light/dark cycle. Mice had *ad libitum* access to food and water. A tumor xenograft mouse model was constructed by subcutaneous injection of the transfected Huh7 cells (1 × 10^6^ cells/200 μL) through the right armpit. The mice were randomly divided into four groups (*n* = 4): (a) normal group, mice were subcutaneously injected with Huh7 cells; (b) LV‐NC group, mice were subcutaneously injected with LV‐NC‐transfected Huh7 cells; (c) LV‐CXCR4 group, mice were subcutaneously injected with LV‐CXCR4‐transfected Huh7 cells; and (d) LV‐CXCR4 + LV‐sh c‐Met group, mice were subcutaneously injected with LV‐CXCR4‐transfected Huh7 cells and LV‐sh c‐Met‐transfected Huh7 cells. When the tumor volume was 100 mm^3^, mice received 45 mg·kg^−1^ gefitinib continuously by gavage.

Ten days after inoculation, the tumor volume of HCC was measured every 5 days. The long and short diameters of the tumor tissues were measured using a vernier caliper; tumor volume (mm^3^) = (length/width^2^)/2. After 30 days, the mice were anesthetized by 5% isoflurane inhalation and then euthanized by cervical dislocation. The tumor tissues were stripped from the mice. The tumors were collected and weighed. All protocols were carried out following the *Guide for the Care and Use of Laboratory Animals* (National Institutes of Health), with the approval of the Ethics Committee of First Affiliated Hospital of Harbin Medical University.

### Gene expression

Total RNA from Huh7 and Huh7‐R cells was extracted using TRIzol reagent (Cat: 15596026; Invitrogen, Carlsbad, CA, USA). The concentration and integrity of RNA was detected using NanoDrop 2000 spectrophotometer (Thermo Fisher Scientific, Waltham, MA, USA) and 1.5% agarose gel electrophoresis. Total RNA was reverse transcribed using PrimeScript™ RT reagent Kit (Perfect Real Time; Cat: RR047A; Takara, Tokyo, Japan) to generate complementary DNA. The relative quantification of genes was assessed by performing quantitative real‐time PCR (qRT‐PCR) applying TB Green® Premix Ex Taq™ II (Tli RNaseH Plus; Cat: RR820A; Takara). The data were analyzed using 2^−∆∆CT^ method.

### Protein expression

The expression of proteins in Huh7 and Huh7‐R cells was detected by performing western blot (WB). Total protein from Huh7 cells was extracted applying Total Protein Extraction Kit (Cat: BC3710; Solarbio) as the protocol described. BCA Protein Assay Kit (Cat: PC0020; Solarbio) was used to assess the protein concentration. Protein samples were electrophoresed on 10% SDS/PAGE gels. Then, the separated proteins were transferred onto polyvinylidene fluoride membranes (Cat: ISEQ00010; Merck Millipore, Billerica, MA, USA). Subsequently, the membranes were incubated with the primary antibodies, *CXCR4* (1 : 1000, Cat: 11073‐2‐AP; Proteintech, Wuhan, China), *Cav‐1* (1 : 1000, Cat: 16447‐1‐AP; Proteintech), p‐Cav‐1 (1 : 2000, Cat: ab75876; Abcam), c‐Met (1 : 1000, Cat: 25869‐1‐AP; Proteintech), p‐c‐Met (1 : 1000, Cat: ab68141; Abcam), Raf‐1 (1 : 1000, Cat: 26863‐1‐AP; Proteintech) and EGFR (1 : 10 000, Cat: 66455‐1‐Ig; Proteintech) at 4 °C for 12 h. After blocking with 5% skim milk, the membranes were incubated with goat anti‐rabbit or goat anti‐mouse horseradish peroxidase‐conjugated secondary antibody (1 : 5000, Cat: SA00001‐2/SA00001‐1; Proteintech). GAPDH antibody (1 : 5000, Cat: 10494‐1‐AP; Proteintech) was used as a reference protein for normalization. The data were analyzed by imagej software (National Institutes of Health, Bethesda, MD, USA).

### Cell proliferation

Cell proliferation was assessed applying Cell Counting Kit‐8 (CCK‐8) assay. Huh7 or Huh7‐R cells were seeded into a 96‐well plate and cultured at 37 °C for 48 h. Then, cells (100 μL) were incubated with 10 μL CCK‐8 reagent (Cat: C0037; Beyotime, Shanghai, China) at 37 °C for 1 h. The absorbance of each well at 450 nm was measured by a microplate reader (Thermo Fisher Scientific).

### Apoptosis

Huh7 cell apoptosis was examined applying Annexin V–FITC/PI Apoptosis Detection Kit (Cat: 40302ES20; YEASEN, Shanghai, China). In brief, Huh7 cells were washed with PBS buffer and mixed with 100 μL 1× Binding Buffer. The cell suspension was stained with Annexin V–FITC (5 μL) and propidium iodide (PI) staining solution (10 μL) at darkness for 15 min. Then, the cell suspension was mixed with 400 μL 1× Binding Buffer on ice. Apoptosis was determined by flow cytometry (BD Biosciences, San Jose, CA, USA) in 1 h.

### Statistical analysis

Each assay was performed three times. All data are reported as mean ± standard deviation. spss 22.0 statistical software (IBM, Armonk, NY, USA) was used for statistical analysis. Two‐tailed Student's *t* test and one‐way ANOVA were used to analyze the statistical difference. A *P* value <0.05 was considered as a significant difference.

## Results

### CXCR4 overexpression promoted gefitinib resistance of Huh7 cells

**Fig. 1:**
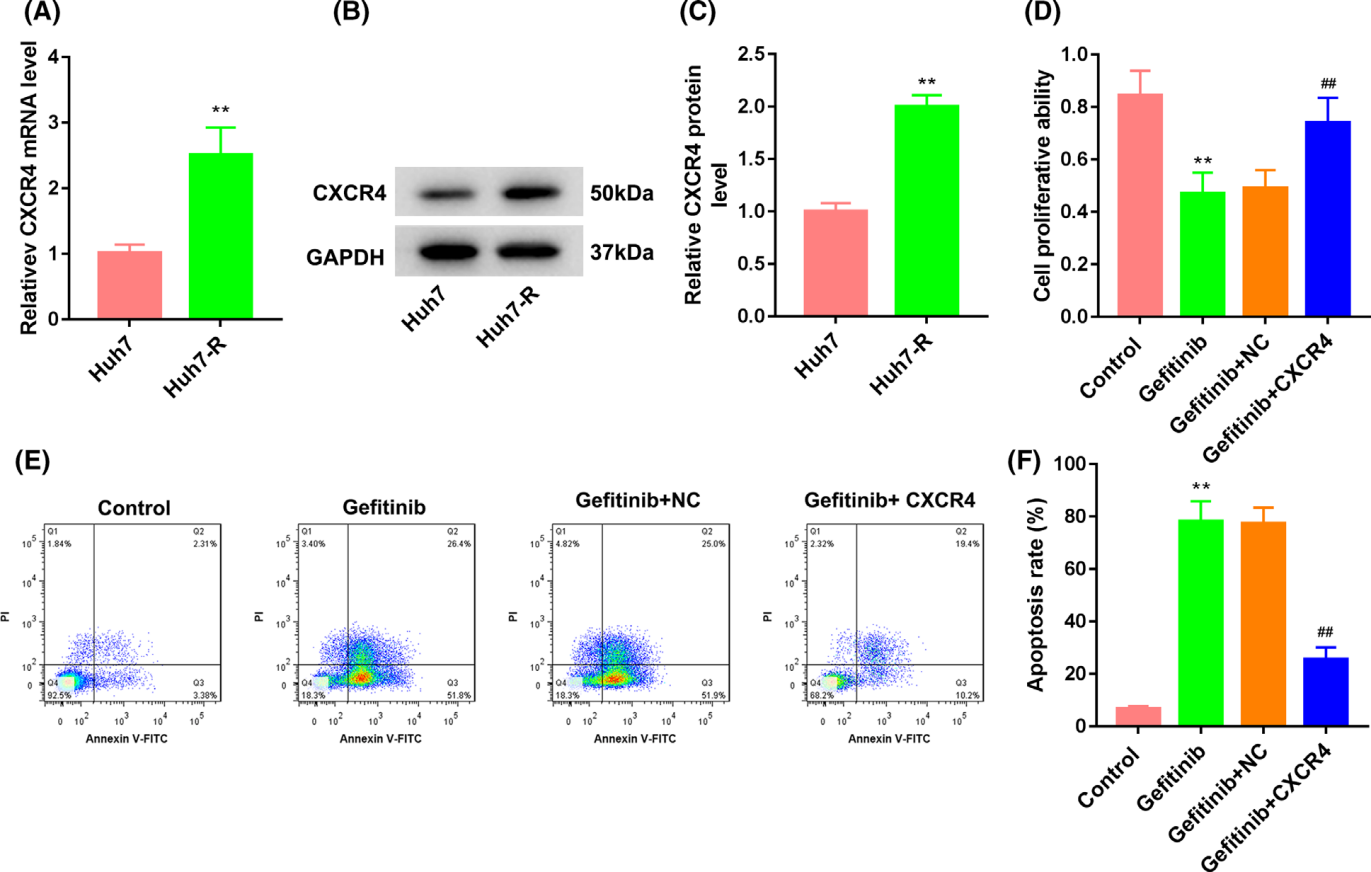
*CXCR4* overexpression promoted proliferation and inhibited apoptosis of Huh7 cells. qRT‐PCR (A) and WB (B, C) were performed to assess the gene and protein expression of *CXCR4* in Huh7 and Huh7‐R cells. *n* = 3. Two‐tailed Student's *t*‐test. Huh7 cells were transfected with pcDNA3.1‐CXCR4 or pcDNA3.1‐NC, followed by treatment of gefitinib. Proliferation and apoptosis of Huh7 cells were examined by CCK‐8 assay (D) and flow cytometry (E, F). *n* = 3. One‐way ANOVA. Data were presented as mean ± standard deviation. **P < 0.01 versus Huh7 or Ctrl; ^##^ *P* < 0.01 versus gefitinib + NC.

To explore the biological role of *CXCR4* in gefitinib resistance of HCC, we compared the expression of *CXCR4* between Huh7 and Huh7‐R cells. qRT‐PCR and WB data showed that *CXCR4* mRNA and protein were highly expressed in Huh7‐R cells with respect to Huh7 cells (Fig. 1A–C). We also found that the expression of p‐c‐Met was significantly increased in Huh7‐R cells as compared with Huh7 cells. However, there was no change in EGFR and c‐Met expression between Huh7 and Huh7‐R cells (Fig. S1A–C). Then, we overexpressed *CXCR4* in Huh7 cells and assessed the impact of *CXCR4* overexpression on cell proliferation and apoptosis in gefitinib‐treated Huh7 cells by CCK‐8 assay and flow cytometry. Cell proliferation of Huh7 cells was severely reduced by gefitinib treatment, which was effectively rescued by *CXCR4* up‐regulation (Fig. 1D). Moreover, apoptosis of Huh7 cells was notably enhanced in the presence of gefitinib. *CXCR4* overexpression partly repressed gefitinib‐induced apoptosis in Huh7 cells (Fig. 1E,F). Furthermore, we silenced *CXCR4* in HuH‐7R cells and measured the influence of *CXCR4* knockdown on the gefitinib resistance of HuH‐7R cells. Gefitinib treatment had no effect on proliferation ability of HuH‐7R cells. *CXCR4* deficiency reduced the proliferation ability of HuH‐7R cells after gefitinib treatment (Fig. S1D). Thus, these data suggested that *CXCR4* overexpression promoted gefitinib resistance of Huh7 cells, and *CXCR4* knockdown reversed gefitinib resistance of HuH‐7R cells.

### CXCR4 overexpression promoted gefitinib resistance of Huh7 cells by activating the c‐Met signaling pathway

**Fig. 2:**
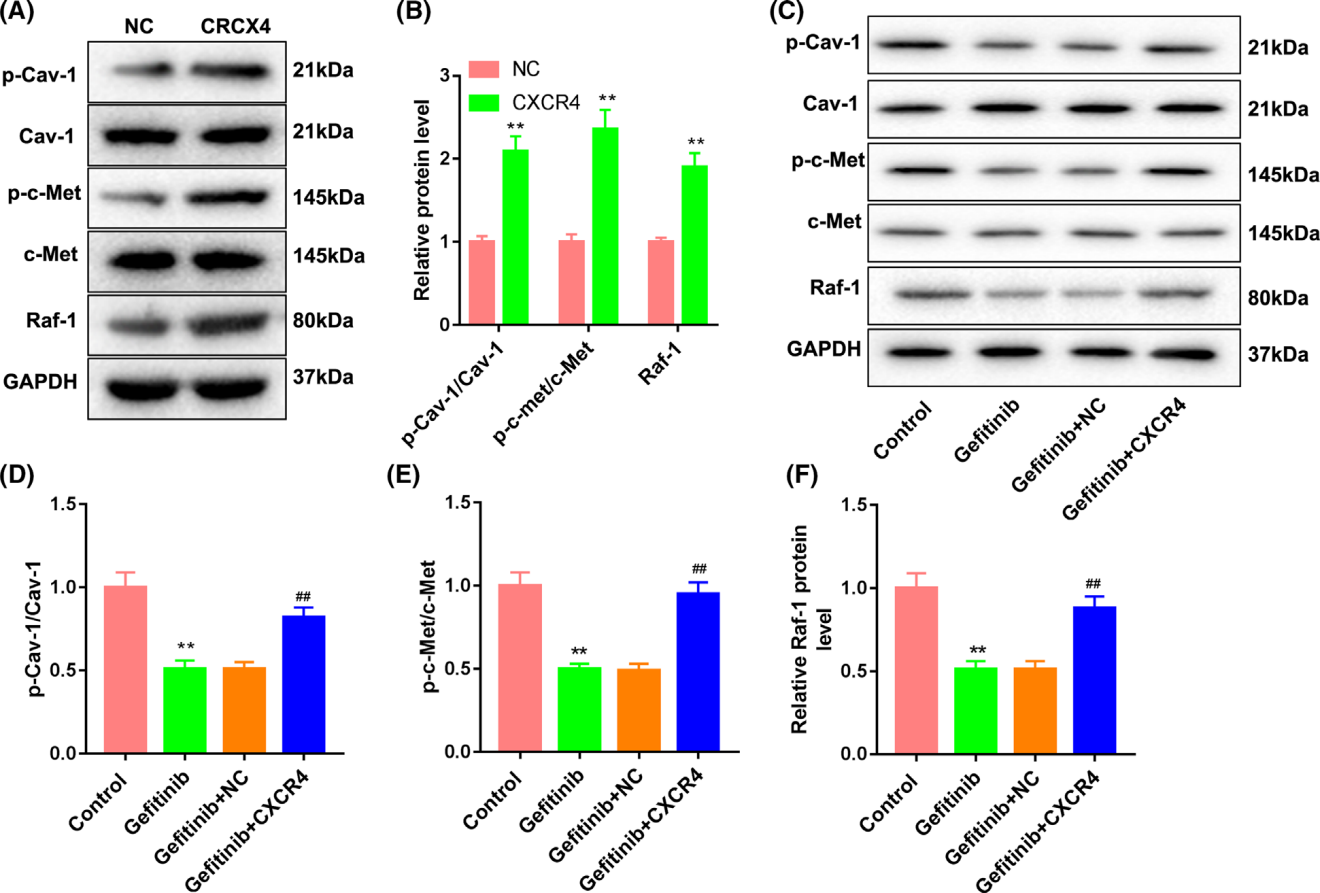
*CXCR4* activated c‐Met and *Cav‐1* signaling pathways. (A, B) Huh7 cells were transfected with pcDNA3.1‐CXCR4 or pcDNA3.1‐NC. WB was performed to assess the expression of *Cav‐1*, p‐Cav‐1, c‐Met, p‐c‐Met and Raf‐1 in Huh7 cells. *n* = 3. Two‐tailed Student's *t*‐test. (C–F) Huh7 cells were transfected with pcDNA3.1‐CXCR4 or pcDNA3.1‐NC, followed by treatment of gefitinib. WB was performed to assess the expression of *Cav‐1*, p‐Cav‐1, c‐Met, p‐c‐Met and Raf‐1 in Huh7 cells. *n* = 3. One‐way ANOVA. Data were presented as mean ± standard deviation. **P < 0.01 versus NC, Ctrl; ^##^ *P* < 0.01 versus gefitinib + NC.

**Fig. 3:**
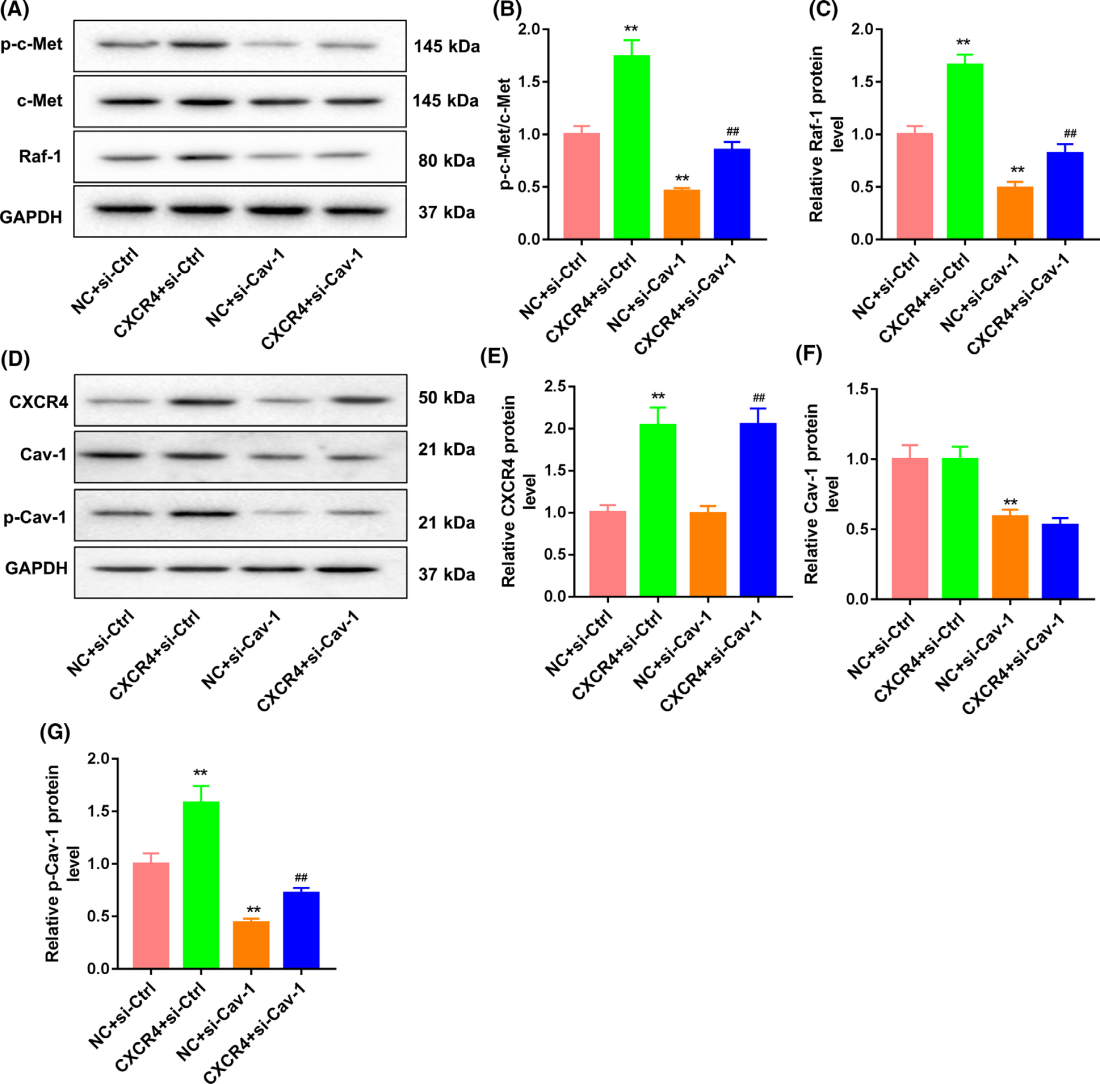
*CXCR4* activated c‐Met via the *Cav‐1* signaling pathway. Huh7 cells were cotransfected with pcDNA3.1‐CXCR4 or pcDNA3.1‐NC and si‐Cav‐1 or si‐Ctrl. (A–C) WB was performed to examine the expression of c‐Met, p‐c‐Met and Raf‐1 in Huh7 cells. (D–G) WB was performed to examine the expression of *CXCR4*, *Cav‐1* and p‐Cav1 in Huh7 cells. *n* = 3. One‐way ANOVA. Data were presented as mean ± standard deviation. **P < 0.01 versus NC + si‐Ctrl; ^##^ *P* < 0.01 versus NC + si‐Cav‐1.

We further investigated the mechanism of action of *CXCR4* in regulating gefitinib resistance of Huh7 cells. WB was performed to examine the impact of *CXCR4* overexpression on the expression of *Cav‐1* and c‐Met signaling pathway‐related proteins in the Huh7 cells. The total expressions of *Cav‐1* and c‐Met were not affected by *CXCR4* overexpression in Huh7 cells. However, the expressions of p‐Cav‐1, p‐c‐Met and Raf‐1 were increased in Huh7 cells after transfection of the *CXCR4* overexpression vector (Fig. 2A,B). Similarly, gefitinib treatment also had no effect on the expression of *Cav‐1* and c‐Met in Huh7 cells. Gefitinib treatment caused a down‐regulation of p‐Cav‐1, p‐c‐Met and Raf‐1 in Huh7 cells, which was effectively abolished by *CXCR4* overexpression (Fig. 2C–F). Furthermore, we determined the influence of *Cav‐1* knockdown on the *Cav‐1* and c‐Met signaling pathway by WB analysis, showing that the expression of c‐Met in Huh7 cells had no changes in the presence of pcDNA3.1‐CXCR4 or si‐Cav‐1. The expressions of p‐c‐Met and Raf‐1 were enhanced by *CXCR4* up‐regulation in Huh7 cells. *Cav‐1* deficiency caused a decrease in the expression of p‐c‐Met and Raf‐1 in Huh7 cells. However, the influence conferred by *CXCR4* up‐regulation was partly rescued by *Cav‐1* silencing (Fig. 3A–C). In addition, *CXCR4* overexpression enhanced the expression of *CXCR4*, and *Cav‐1* deficiency reduced *Cav‐1* expression in Huh7 cells. Importantly, *CXCR4* overexpression had no influence on *Cav‐1* expression; similarly, *Cav‐1* silencing did not cause a substantive change in *CXCR4* expression. *CXCR4* overexpression caused an up‐regulation of p‐Cav‐1, and *Cav‐1* silencing led to a down‐regulation of p‐Cav‐1 in Huh7 cells. Inhibition of *Cav‐1*‐mediated down‐regulation of p‐Cav‐1 was rescued by *CXCR4* overexpression (Fig. 3D–G).

**Fig. 4:**
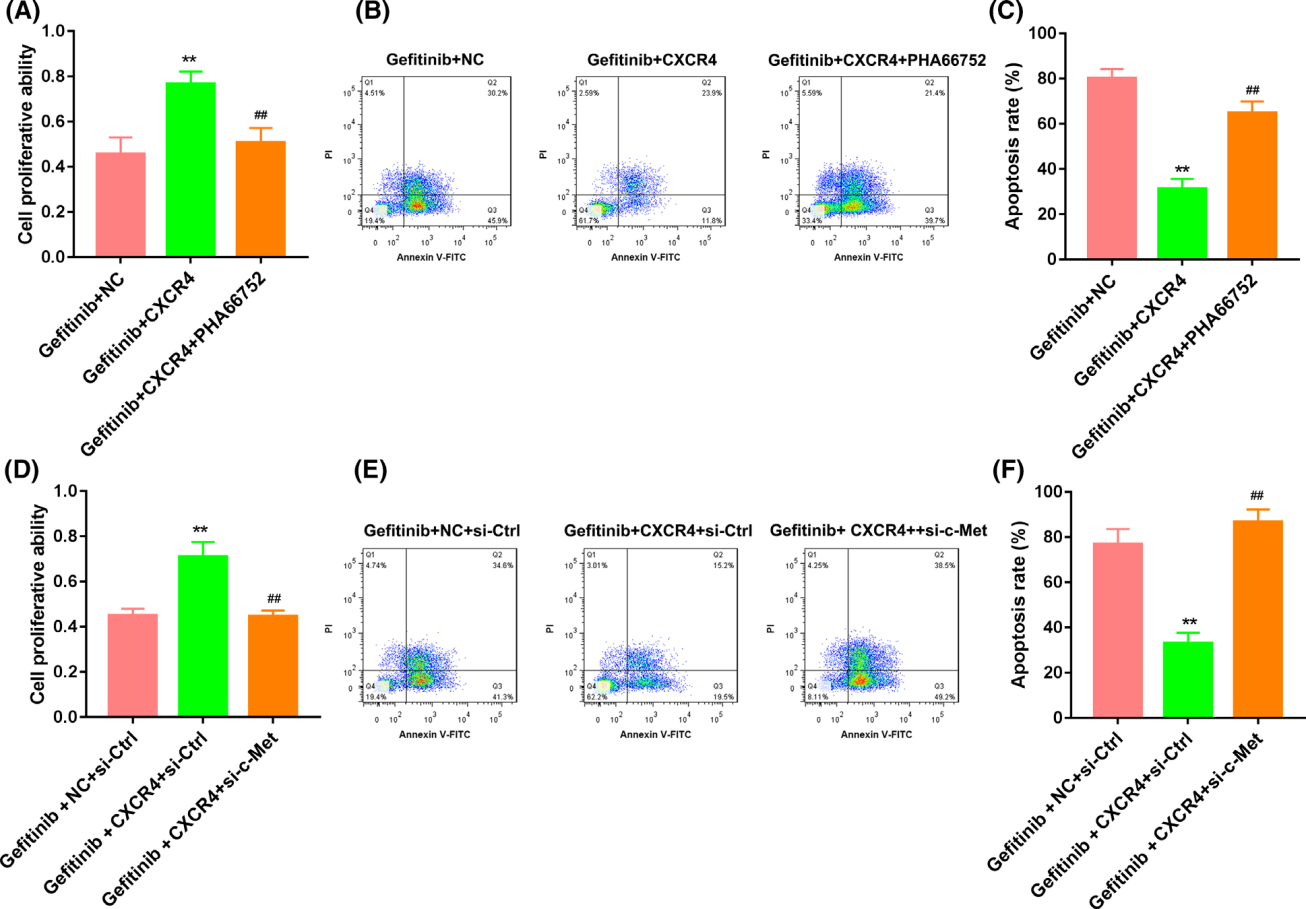
*CXCR4* overexpression promoted proliferation and inhibited apoptosis of Huh7 cells by activating the c‐Met signaling pathway. Huh7 cells were transfected with pcDNA3.1‐CXCR4 or pcDNA3.1‐NC and then treated with gefitinib or combined with PHA‐665752. Proliferation and apoptosis of Huh7 cells were examined by CCK‐8 assay (A) and flow cytometry (B, C). Huh7 cells were cotransfected with pcDNA3.1‐CXCR4 or pcDNA3.1‐NC and si‐c‐Met or si‐Ctrl and then treated with gefitinib. CCK‐8 assay (D) and flow cytometry (E, F) were performed to detect proliferation and apoptosis of Huh7 cells. *n* = 3. One‐way ANOVA. Data were presented as mean ± standard deviation. **P < 0.01 versus gefitinib + NC or gefitinib + NC + si‐Ctrl; ^##^ *P* < 0.01 versus gefitinib + *CXCR4* or gefitinib + *CXCR4* + si‐Ctrl.

Next, Huh7 cells were treated with PHA‐665752 to inhibit c‐Met activity. The results obtained from CCK‐8 assay and flow cytometry showed that *CXCR4* overexpression enhanced proliferation and inhibited apoptosis of Huh7 cells, which was effectively rescued by PHA‐665752 treatment (Fig. 4A–C). We also found that the expressions of p‐c‐Met and Raf‐1 were significantly increased by *CXCR4* overexpression in the gefitinib‐treated Huh7 cells. PHA‐665752 treatment reversed *CXCR4* overexpression‐mediated promotion of p‐c‐Met and Raf‐1 expression (Fig. S2A–C). Furthermore, *CXCR4* overexpression caused an increase in proliferation and led to a decrease in apoptosis of Huh7 cells. Knockdown of c‐Met effectively rescued the influence of *CXCR4* overexpression on proliferation and apoptosis of Huh7 cells (Fig. 4D–F). In addition, *CXCR4* overexpression enhanced p‐c‐Met and Raf‐1 expression in gefitinib‐treated Huh7 cells. *CXCR4* overexpression‐mediated promotion of Raf‐1 expression was repressed by c‐Met knockdown (Fig. 2D–F).

Taken together, these findings demonstrated that *CXCR4* overexpression activated c‐Met via the *Cav‐1* signaling pathway, thereby promoting gefitinib resistance of Huh7 cells.

### CXCR4 overexpression promoted tumor growth of HCC in mice by activating the c‐Met signaling pathway

**Fig. 5:**
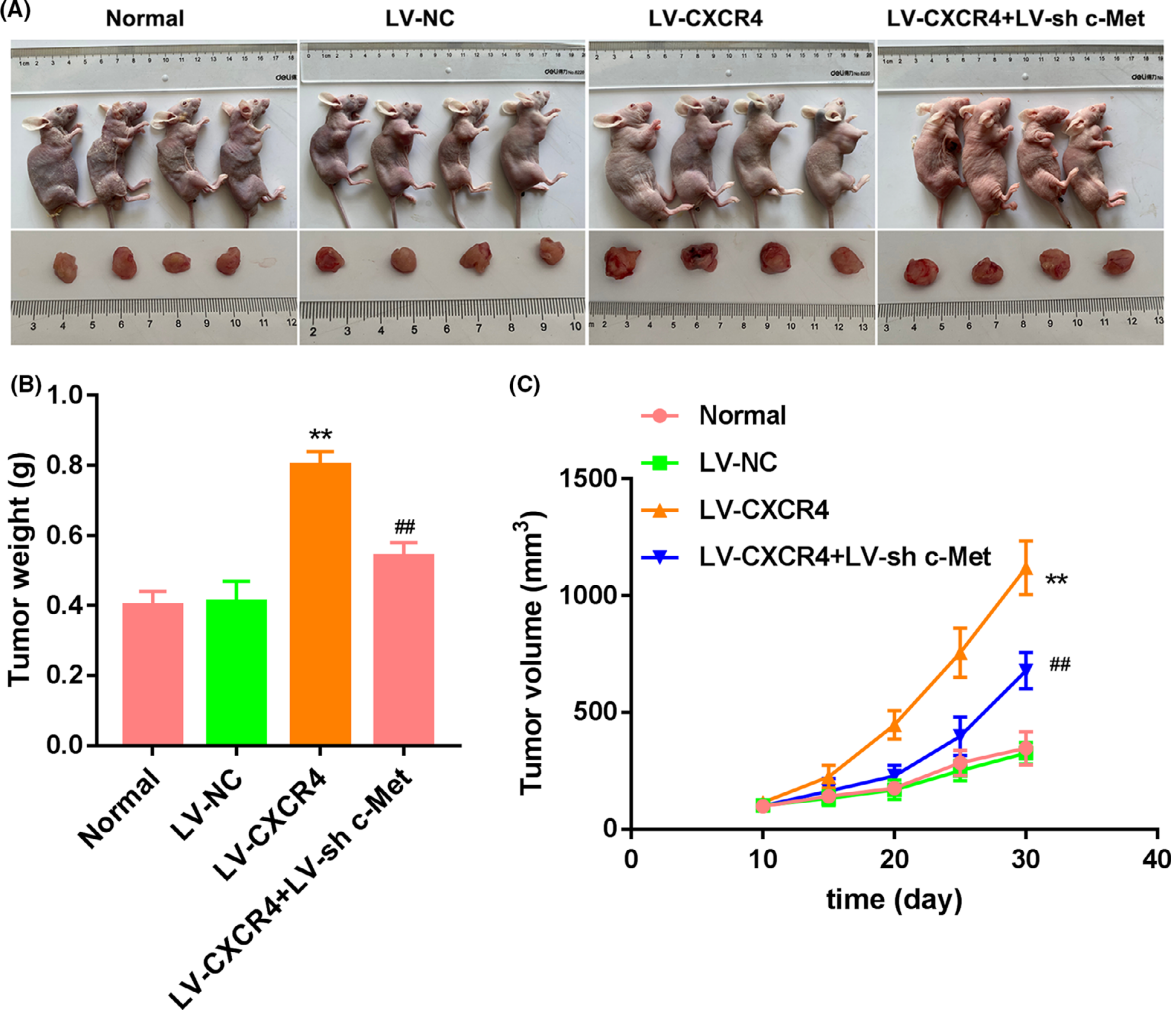
*CXCR4* overexpression promoted tumor growth of HCC in mice via the c‐Met signaling pathway. (A) The tumor xenograft mouse model was constructed by subcutaneous injection of the normal or transfected Huh7 cells and received administration of gefitinib. The weight (B) and volume (C) of tumor in mice were measured. *n* = 4. One‐way ANOVA. Data were presented as mean ± standard deviation. **P < 0.01 versus normal; ^##^ *P* < 0.01 versus LV‐CXCR4.

Finally, we constructed a tumor xenograft model by injection of Huh7 cells after *CXCR4* overexpression or combined with c‐Met knockdown to verify the impact of *CXCR4* on tumor growth in HCC. LV‐CXCR4‐transfected Huh7 cells of inoculation significantly enhanced the weight and volume of tumor in mice. However, the weight and volume of tumor in mice were notably decreased after inoculation of LV‐CXCR4 + LV‐sh c‐Met‐transfected Huh7 cells (Fig. 5A–C). Thus, *in vivo* results confirmed that *CXCR4* overexpression promoted tumor growth of HCC in mice by activating the c‐Met signaling pathway.

## Discussion

Gefitinib is one of the important new molecular alternative drugs developed in recent years. Gefitinib is a kind of EGFR‐TKI drug that has a synergistic effect on chemotherapy drugs [15, 16]. The inhibitory effect of gefitinib on tumors may be related to block the cell cycle, promote cell apoptosis and inhibit the secretion of vascular endothelial growth factor and angiopoiesis. In addition, gefitinib also can be used to inhibit the growth of HCC cells and tumor growth in mice [17, 18]. Moreover, a previous study has reported that *CXCR4* is associated with gefitinib resistance in cancer [19]. Gefitinib promotes migration and epithelial‐to‐mesenchymal transition of lung adenocarcinoma cells harboring EGFR mutation by enhancing *CXCR4* expression. However, the occurrence of gefitinib resistance in patients with HCC is the main reason for failure of tumor treatment. In our study, we investigated the molecular mechanism of *CXCR4* in gefitinib resistance in HCC. We found that *CXCR4* and p‐c‐Met were highly expressed in Huh7‐R cells. *CXCR4* overexpression promoted proliferation and inhibited apoptosis of Huh7 cells in the presence of gefitinib. *CXCR4* knockdown reversed gefitinib resistance of HuH‐7R cells. These data suggested that *CXCR4* and c‐Met were associated with the occurrence of gefitinib resistance in HCC.

*CXCR4* has been reported to be associated with the progression of HCC. *CXCR4* participates in promoting migration and invasion of HCC cells. Cordycepin, a traditional Chinese medicine extract, causes down‐regulation of *CXCR4*, thereby inhibiting the migration and invasion of human HCC cells [20]. Benedicto *et al*. [21] have confirmed that the *CXCL12*/*CXCR4* signaling pathway plays a crucial role in promoting liver metastasis. AMD3100 inhibits *CXCR4* activity and blocks the *CXCL12*/*CXCR4* axis, thereby inhibiting the contribution of both stromal and cancer cells to liver metastasis. Celastrol inhibits proliferation and migration of HCC cells by inhibiting the *CXCR4* signaling pathway to impede the progression of HCC [22]. Our work revealed that gefitinib inhibited *Cav‐1* and c‐Met signaling pathways, which were rescued by *CXCR4* overexpression. Moreover, *CXCR4* overexpression had no influence on *Cav‐1* expression; similarly, *Cav1* silencing did not cause a substantive change in *CXCR4* expression. However, *CXCR4* up‐regulation activated *Cav‐1* and c‐Met signaling pathways by promoting the expression of p‐Cav‐1 and p‐c‐Met, which was abolished by *Cav‐1* silencing. Thus, *CXCR4* may promote the activation of c‐Met through the *Cav‐1* signaling pathway. In addition, PHA‐665752 blocked c‐Met activity, repressed proliferation and enhanced apoptosis of Huh7 cells. The effect of *CXCR4* overexpression on proliferation and apoptosis of Huh7 cells was abolished by c‐Met deficiency. Thus, these findings demonstrated that *CXCR4* overexpression activated c‐Met via the *Cav‐1* signaling pathway, thereby promoting gefitinib resistance of Huh7 cells.

Many studies have confirmed the role of c‐Met in the procession of HCC. *P‐Rex1* promotes proliferation and migration of HCC cells and enhances xenograft tumor growth *in vivo* by activating the HGF/c‐Met signaling pathway [23]. The HGF/c‐Met signaling pathway participates in the liver metastasis of colorectal cancer in mice [24]. Consistently, we verified the effect of *CXCR4* on gefitinib resistance in HCC *in vivo*. *CXCR4* overexpression significantly promoted tumor growth of HCC in mice. However, c‐Met deficiency notably suppressed tumor growth of HCC. Thus, *CXCR4* overexpression promotes tumor growth of HCC by activating the c‐Met signaling pathway. Of course, this article also has some shortcomings. This article studied only the impact of *CXCR4* on the tolerance of Huh7 cells to gefitinib, and whether *CXCR4* had the same effect on other HCC cell lines still needed further study. We will conduct research on this topic in future work.

Previous study has confirmed that the positive crosstalk between *CXCR4* and EGFR increases the migration ability in gastric cancer [25]. In lung cancers, c‐Met amplification induces gefitinib resistance by regulating the phosphorylation of the epidermal growth factor receptor (ERBB3)/phosphatidylinositol 3‐kinase (PI3K) axis [26]. *CXCR4* promotes the phosphorylation of c‐Met and then induces epithelial‐to‐mesenchymal transition and accelerates migration in gastric cancer cells [11]. Consistently, this work also confirmed the carcinogenic effects of *CXCR4* in HCC. *CXCR4* overexpression accelerated the invasive phenotype in HCC through the *Cav‐1*/c‐Met signaling pathway and thus promoted gefitinib resistance of HCC.

In conclusion, our work demonstrates the mechanism of action of *CXCR4* in gefitinib resistance of HCC. *CXCR4* overexpression activates c‐Met via the *Cav‐1* signaling pathway, thereby promoting gefitinib resistance of HCC. Thus, this study highlights novel insights into the mechanism of gefitinib resistance of HCC, and *CXCR4* may be a potential target for HCC treatment.

## Conflict of interest

The authors declare no conflict of interest.

## Author contributions

DZ conceived and designed the project; DZ, ZY, CC, ZZ, YY and ZL acquired the data; DZ and ZL analyzed and interpreted the data; DZ wrote the paper. All authors read and approved the paper.

## Supporting information


**Fig. S1**. The expression of EGFR, c‐Met and p‐c‐Met in Huh7 and Huh7‐R cells. (A–C) WB was performed to assess the expression of EGFR, c‐Met and p‐c‐Met in Huh7 and Huh7‐R cells. *n* = 3. Two‐tailed Student's *t* test. (D) Huh7‐R cells were transfected with si‐CXCR4 or si‐Ctrl, followed by gefitinib treatment. CCK‐8 assay was performed to assess cell proliferation of Huh7‐R cells. *n* = 3. One‐way ANOVA. Data were presented as mean ± standard deviation. **P < 0.01 vs Huh7; **P < 0.01 vs gefitinib + si‐Ctrl. CCK‐8, Cell Counting Kit‐8; *CXCR4*, C‐X‐C chemokine receptor type 4; Ctrl, control; EGFR, epidermal growth factor receptor; NC, negative control; WB, western blot.

**Fig. S2**. *CXCR4* overexpression activated c‐Met and enhanced the expression of Raf‐1 in Huh7 cells. (A–C) Huh7 cells were transfected with pcDNA3.1‐CXCR4 or pcDNA3.1‐NC, and then treated with gefitinib or combined with PHA‐665752. WB was performed to assess the expression of c‐Met, p‐c‐Met and Raf‐1 in Huh7 cells. (D–F) Huh7 cells were co‐transfected with pcDNA3.1‐CXCR4 or pcDNA3.1‐NC and si‐c‐Met or si‐Ctrl, and then treated with gefitinib. WB was performed to assess the expression of c‐Met, p‐c‐Met and Raf‐1 in Huh7 cells. *n* = 3. One‐way ANOVA. Data were presented as mean ± standard deviation. *P < 0.05 vs Gefitinib + NC or Gefitinib + NC + si‐Ctrl; ^#^ *P* < 0.05 vs Gefitinib + *CXCR4* or Gefitinib + *CXCR4* + si‐Ctrl. *CXCR4*, C‐X‐C chemokine receptor type 4; NC, negative control; WB, western blot.

## Data Availability

The data that support the findings of this study are available from the corresponding author upon reasonable request.
