# Supplementary figures and images for: CXCR4 promotes gefitinib resistance of Huh7 cells by activating the c‐Met signaling pathway
Source: FEBS Open Bio. 2021 Oct 19;11(11):3115–25. doi: 10.1002/2211-5463.13305 (PMC8564344; doi:10.1002/2211-5463.13305)

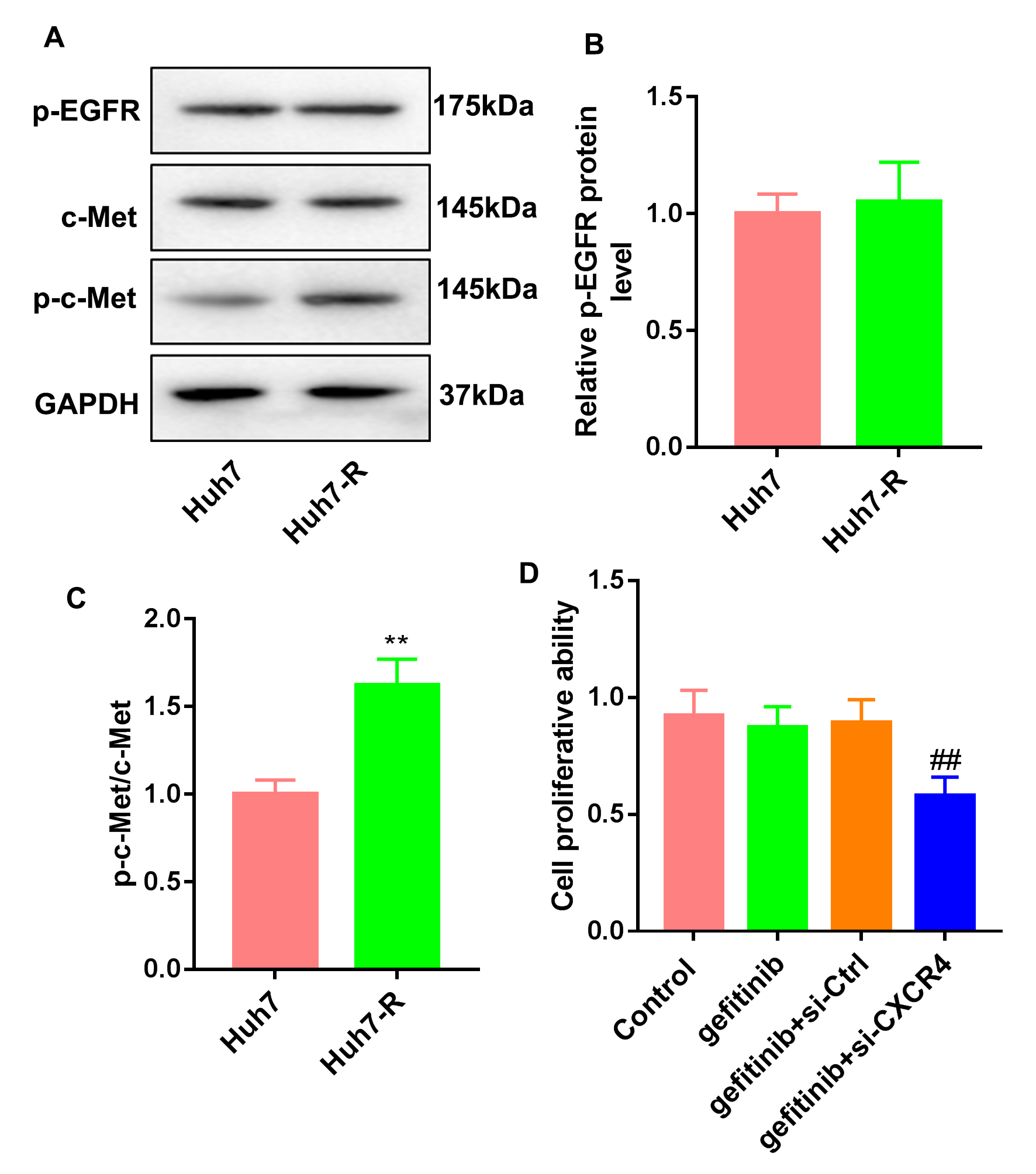

Supplement: Supplementary file 1 — Fig. S1. The expression of EGFR, c‐Met and p‐c‐Met in Huh7 and Huh7‐R cells. (A–C) WB was performed to assess the expression of EGFR, c‐Met and p‐c‐Met in Huh7 and Huh7‐R cells. n = 3. Two‐tailed Student's t test. (D) Huh7‐R cells were transfected with si‐CXCR4 or si‐Ctrl, followed by gefitinib treatment. CCK‐8 assay was performed to assess cell proliferation of Huh7‐R cells. n = 3. One‐way ANOVA. Data were presented as mean ± standard deviation. **P < 0.01 vs Huh7; **P < 0.01 vs gefitinib + si‐Ctrl. CCK‐8, Cell Counting Kit‐8; CXCR4, C‐X‐C chemokine receptor type 4; Ctrl, control; EGFR, epidermal growth factor receptor; NC, negative control; WB, western blot. [file FEB4-11-3115-s002.tif]

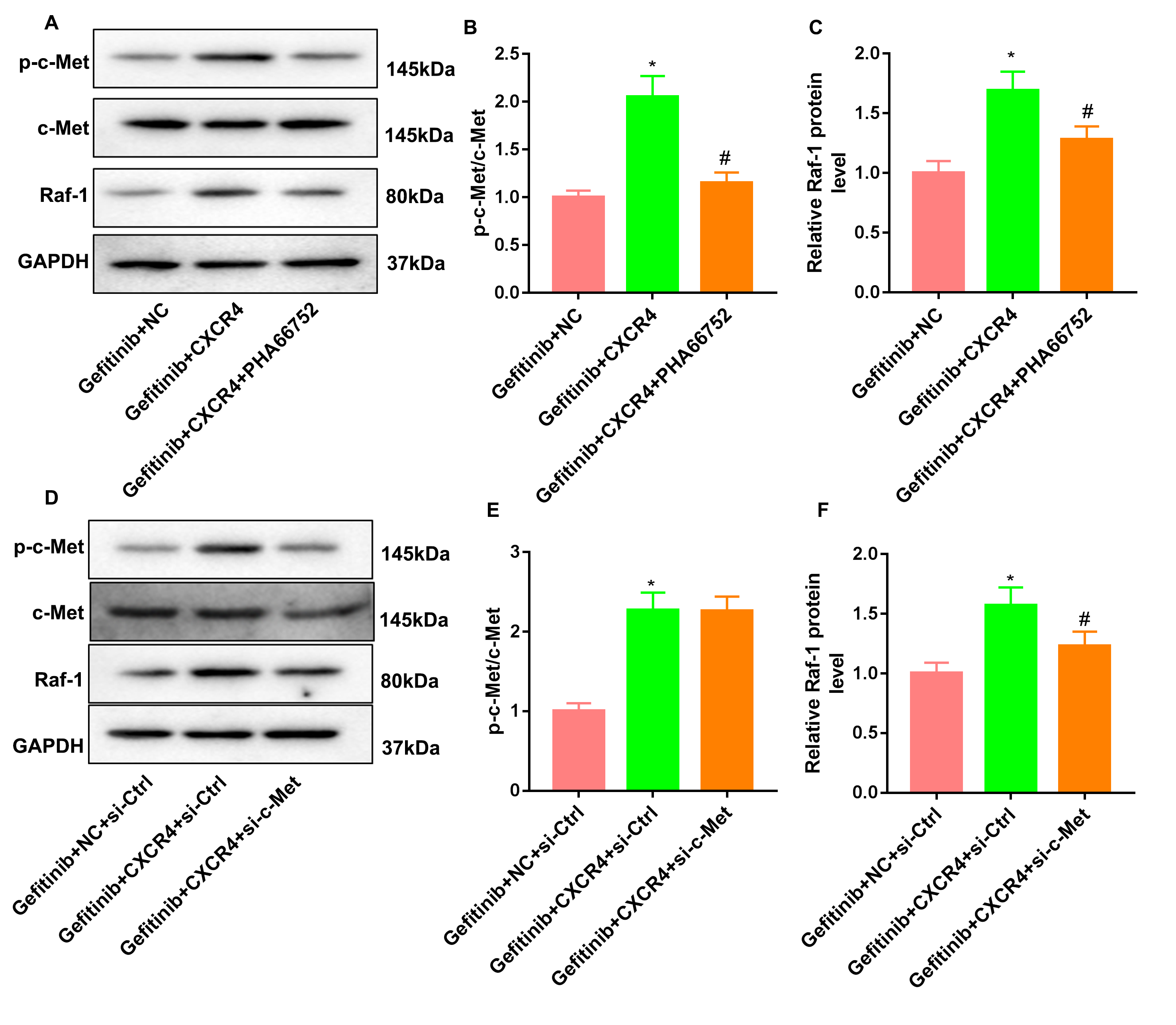

Supplement: Supplementary file 2 — Fig. S2. CXCR4 overexpression activated c‐Met and enhanced the expression of Raf‐1 in Huh7 cells. (A–C) Huh7 cells were transfected with pcDNA3.1‐CXCR4 or pcDNA3.1‐NC, and then treated with gefitinib or combined with PHA‐665752. WB was performed to assess the expression of c‐Met, p‐c‐Met and Raf‐1 in Huh7 cells. (D–F) Huh7 cells were co‐transfected with pcDNA3.1‐CXCR4 or pcDNA3.1‐NC and si‐c‐Met or si‐Ctrl, and then treated with gefitinib. WB was performed to assess the expression of c‐Met, p‐c‐Met and Raf‐1 in Huh7 cells. n = 3. One‐way ANOVA. Data were presented as mean ± standard deviation. *P < 0.05 vs Gefitinib + NC or Gefitinib + NC + si‐Ctrl; # P < 0.05 vs Gefitinib + CXCR4 or Gefitinib + CXCR4 + si‐Ctrl. CXCR4, C‐X‐C chemokine receptor type 4; NC, negative control; WB, western blot. [file FEB4-11-3115-s001.tif]
